# Astaxanthin Alleviates Inflammatory Response in Neonatal Necrotizing Enterocolitis Rats by Regulating NOD2/TLR4 Pathway

**DOI:** 10.1155/2023/6078308

**Published:** 2023-03-27

**Authors:** Xuandong Zhang, Yujia Luo, Rui Gu, Zhou Jiang

**Affiliations:** Department of Neonatology, Run Run Shaw Hospital, Zhejiang University School of Medicine, Hangzhou, Zhejiang Province, China

## Abstract

**Background:**

Necrotizing enterocolitis (NEC) is often associated with exaggerated activation of inflammatory response. Astaxanthin has been shown in studies to have a positive and advantageous effect on anti-inflammatory response. Hence, it is of great significance to study the protective effect of astaxanthin in NEC disease and its molecular mechanism.

**Objective:**

The present study was to investigate whether astaxanthin attenuates NEC rats and to explore its potential mechanism. *Material and Methods.* Hematoxylin-eosin staining was used to observe the pathological change of the intestinal tissue in NEC rats. Subsequently, we determined the anti-oxidative stress, anti-apoptosis, and anti-inflammation in astaxanthin with enzyme-linked immunosorbent assay kits, TUNEL staining, western blot, and immunohistochemistry assay. Furthermore, we added nucleotide-binding oligomerization domain 2 (NOD2) inhibitor to certify the molecular pathway of the astaxanthin in NEC rats.

**Results:**

Astaxanthin improved the pathological changes of the intestinal tissues. It restrained inflammation, oxidative stress, and protected cells from apoptosis in the intestinal tissue and serum of the NEC rats. Moreover, astaxanthin enhanced NOD2, whereas it suppressed toll-like receptor 4 (TLR4), nuclear factor-*κ*B (NF-*κ*B) pathway-related proteins. Apart from that, the NOD2 inhibitor offset the protective effect of the astaxanthin towards the NEC rats.

**Conclusion:**

The present study indicated that astaxanthin alleviated oxidative stress, inflammatory response, and apoptosis in NEC rats by enhancing NOD2 and inhibiting TLR4 pathway.

## 1. Introduction

In preterm newborns, necrotizing enterocolitis (NEC) is a dangerous illness that can be fatal. The frequency of NEC is rising despite clinical advancements [1]. With a high mortality rate (20–30%), severe NEC frequently comes with the intestinal wall, perforation necrosis, and peritonitis [2]. For a long time, it was believed that multiple risk factors, including preterm, enteral feeding, intestinal ischemia, and bacterial impacts, contributed to the pathogenesis of NEC [3], eventually leading to inflammation and necrosis.

Inflammation response is crucial in the pathogenesis of NEC in neonates [4]. The innate immune receptor toll-like receptor 4 (TLR4) appears to play a central role in the inflammation of pathogenesis in NEC. Excessive signaling in the epithelial TLR4 pathway in response to lipopolysaccharide (LPS) leads to enterocyte apoptosis resulting in enterocyte loss and subsequent delayed repair through inhibition of migration and TLR4-mediated loss of intestinal stem cells [5]. These factors lead to bacterial and LPS translocation into the circulation, which results in pro-inflammatory cytokine production, reactive oxygen species (ROS) production, increased expression of inducible nitric oxide synthase (iNOS), and TLR4-mediated loss and dysregulation of endothelial nitric oxide synthase (eNOS) leading to impaired perfusion [6]. Likewise, nucleotide-binding oligomerization domain 2 (NOD2) is an important regulator of resistance to microbial invasion and maintenance of tissue homeostasis in intestinal diseases of the organism [7]. It has been shown that the signaling pathways of TLR4 and NOD2 can regulate each other in inflammatory cells [7]. Another study mentioned that there was an interaction between NOD2 and TLR4 receptors in enterocytes, which drives the extent of intestinal damage in the pathogenesis of NEC. They demonstrated that NOD2 activation as well as expression of the NOD2 protein itself limits the extent of TLR4 signaling in enterocytes. This mechanism links TLR4 activation to cell death and can be constrained by NOD2 activity [8].

Astaxanthin is a type of carotene found in crabs, algae, and many other seafoods. The majority of natural astaxanthin today is derived from *Streptococcus sanguinis*, which is more stable than synthetic astaxanthin. Due to its potent effects on oxidation resistance, fatigue resistance, inflammation resistance, and immune regulation, natural astaxanthin is well-liked in the functional food sector [9–12]. Astaxanthin exerted a function to reduce inflammation and improve gut microbiota in hepatopathy mice. Furthermore, the study reported that feeding mice astaxanthin-rich yeast for 21 days increased antibody expression in weaned mice's jejunum and ileum [13]. In addition, in cecum injury and inflammation of mice, astaxanthin regulates the diversity of cecal microbiota and the TLR4 signaling pathway [14]. Sequence-targeted astaxanthin nanoparticles can significantly alleviate inflammation in colitis by modulating the TLR4 signaling pathway [15]. However, it remains unclear how astaxanthin exerted an effect on intestinal function in NEC.

Hence, in our study, we hypothesized that astaxanthin could attenuate intestinal injury in a rat model of NEC through increasing NOD2, inhibiting the TLR4 signaling pathway to exert anti-oxidative stress, anti-inflammatory, and anti-apoptotic function.

## 2. Materials and Methods

### 2.1. Animal Treatment and Group Administration

Zhejiang Eyong Pharmaceutical Research and Development Center's Animal Center's Ethics Committee approved the animal research. Animal use license number: SYXK (Zhe) 2021-0033. Sprague-Dawley (SD) rats were purchased from Zhejiang Weitong Lihua Laboratory Animal Technology Co., LTD., Animal Production License No: SCXK (Zhe) 2019-0001. On the 14th day of pregnancy, a caesarean surgery was performed on the rats to remove preterm SD rats, both male and female.

The rats were divided into three groups: the normal group, the NEC group, and the NEC + Astaxanthin group (*n* = 8 in each group). As previously reported [16], neonatal SD rats in 2 hours of birth were treated with artificial feeding and were stimulated with cold under hypoxic conditions as NEC model. Intragastric administration of rat milk substitutes was used to feed neonatal rats. After that, the rats were subsequently subjected to 100% nitrogen hypoxia for 2 minutes, followed by stimulation of 10 minutes with cold condition at 4°C (twice a day for 3 days). It was considered a successful NEC model when rats showed excretion of yellow-green mucus to varying degrees and other NEC clinical symptoms. Astaxanthin (Sigma-Aldrich; Merck KGaA) which was mixed in olive oil and milk was administered by intragastric twice a day of 60 mg/kg/day by gavage with NEC rats for 4 days in NEC + Astaxanthin group.

Based on the previous experiments, we added NEC + Subsequently, Astaxanthin was administered by intragastric (60 mg/kg twice daily for 4 days) to NEC rats. NOD2 inhibitor (NOD-IN-1, HY-100691, MedChemExpress company) 13.8 mg/kg was injected intraperitoneally in rats once daily for 3 days before exposure to NEC according to the previous study [17]. Subsequently, astaxanthin (60 mg/kg twice daily for 4 days) was administered by intragastric to NEC rats.

### 2.2. Sample Collection

CO_2_ inhalation was used to euthanize all rats on the sixth day after modeling. After intestinal tissues from the duodenum to the anus were removed, a 1 cm sample of the proximal ileocecal intestine was collected. The terminal ileum tissues were also paraffin-embedded, washed, fixed, and cut into serial sections that were 5 *μ*m thick for further research.

### 2.3. TUNEL Staining

Deparaffinized tissue sections were incubated with proteinase K (G1205, Servicebio) in a humidified chamber for 15 minutes, and sections were then incubated with 3% H_2_O_2_ for 10 minutes and terminal deoxynucleotidyl transferase (TdT) (G1507, Servicebio) labeling buffer at 37°C for 1 hour. Then added horseradish peroxidase (HRP) labeled Streptavidin (Streptavidin-HRP) at 37°C for 30 minutes. Finally, under the catalysis of HRP, the apoptotic cells were displayed by 3,3′-Diaminobenzidine (DAB) color, and the apoptotic cells were observed and counted by ordinary light microscope. The positive cell rate is calculated by the number of TUNEL-positive cells/total cells in the visual field.

### 2.4. Enzyme-Linked Immunosorbent Assay (ELISA) Measurement

The blood was centrifuged at 3500 r/min for 15 minutes and supernatant was taken. Interleukin-1*β* (IL-1*β*) (MM-0047R1), interleukin 6 (IL-6) (MM-0190R2), tumor necrosis factor-*α* (TNF-*α*) (MM-0180R2), and macrophage chemoattractant protein-1(MCP-1) (MM-0099R2) ELISA kits (Meimian, Jiangsu, China) were applied to evaluate the level of IL-1*β*, IL-6, TNF-*α*, and MCP-1 in serum.

And the intestinal tissue was removed quickly for further ELISA test, and the blood stain was washed with normal saline at 4°C. The purified water was absorbed by filter paper, and 100 mg of intestinal tissue was cut to make 10% homogenate. Total protein concentrations were measured using bicinchoninic acid (BCA) method. Malondialdehyde (MDA) (Jiancheng, A003-1-2) and superoxide dismutase (SOD) (Jiancheng, A001-3-2) of the intestinal tissue were tested following the manufacturer's instructions. Finally, the optical density of the ELISA microplate was read at 450 nm using a microplate reader.

### 2.5. Hematoxylin-Eosin (H&E) Staining

The intestinal tissues were dehydrated by gradient ethanol and xylene, then immersed in wax. The tissues were affixed to the anti-peeling slides. The slices were baked at 60°C for 12 hours, dewaxed and hydrated by xylene and gradient ethanol, and stained with Hematoxylin and Eosin Stain (Servicebio, G1003). At last, ethanol with low to high concentration was added to dehydrate. Vitrification by xylene and the slices were sealed with neutral balsam, subsequently, the figures of slices were captured with Nikon Eclipse E100.

The area of inflammatory cell infiltration was scored against the degree of the lesion. No inflammation and no lesions score 0; a small number of inflammatory cells infiltrate, lesion invasion, and submucosa score 1; obvious inflammatory cell infiltration, lesion invasion into the muscle layer, and visible recess destruction score 2; obvious inflammatory cell infiltration, lesion invasion into the muscle layer, and obvious destruction of recess score 3; a large number of inflammatory cells infiltrated, lesions invaded the serous layer, and all crypts and epithelium destroyed score 4.

### 2.6. Immunofluorescence Staining

The intestinal tissues were made into paraffin section, and dewaxed with xylene, then ethanol was successively added with high to low concentration to rehydrate the tissue, and antigen repair solution was added, then the sections were incubated in 5% BSA (G5001, SERVICEBIO), 0.2% Triton X-100 and PBS (G0002, SERVICEBIO) to permeabilize the tissue sections. Followed, the sections were incubated with the BAX (AF0120, 1 : 200, Affinity) and Bcl-2 (AF6139, 1 : 200, Affinity) overnight at 4°C, then incubated with Goat Anti-Rabbit IgG (H + L) Fluor488-conjugated (S0018, Affinity) at room temperature for 0.5 hours. Then washed away the antibody, and the DAPI (G1012, SERVICEBIO) was added to stain the cell nuclear. The NIKON ECLIPSE C1 was used to observe and take pictures. ImageJ software was used to analyze the BAX and Bcl-2 protein fluorescence intensity.

### 2.7. Immunohistochemistry (IHC) Assay

The prepared paraffin sections of intestinal tissues were dewaxed with xylene, then ethanol was successively added with high to low concentration to rehydrate the tissue, and antigen repair solution was added, then the sections were washed with hydrogen peroxide to block endogenous peroxidase, later sealed with bovine serum, and incubated overnight with transforming growth Caspase 3 (DF6020, Affinity), at 4°C. On the second day, the corresponding secondary antibody of HRP was incubated. DAB (SERVICEBIO, G1212) was added. The positive expression of DAB was brown-yellow, and the nuclei were stained with hematoxylin. Finally, ethanol with low to high concentration was added to dehydrate, vitrification by xylene, and the slices were added with neutral balsam. The average of density of Caspase 3 was calculated in the visual field, and ImageJ software was used to process data.

### 2.8. Western Blot

Pure protein was extracted from intestinal tissues being cut, homogenized in a cracking buffer, and centrifuged. And the supernatant (protein) after centrifugation was measured with BCA kit (PC0020, Solarbio). The total protein was separated by electrophoresis, and the corresponding protein was transferred to membrane. The non-specific antigen was blocked with 5% milk, and the protein was incubated with the target antibody TNF-*α* (AF7014), iNOS (AF0199), nuclear factor-*κ*B (NF-*κ*B) (AF5006), B-cell lymphoma-2 (Bcl-2) (AF6139), Bcl-2-associated X (BAX) (AF0120), NOD2 (DF12125), TLR4 (AF7017), MyD88 (AF5195), *β*-actin (AF7018) all from Affinity company. After incubation at 4°C for 14–16 hours, unbound antibodies were washed, and the protein was incubated with the corresponding secondary antibodies (Anti-rabbit IgG, HRP-linked Antibody, CST, 7074). Finally, the figure was captured in enhanced chemiluminescence (ECL) imager. ImageJ software was used to calculate the optical density of the target protein strip, and *β*-actin was used as an internal reference.

### 2.9. Statistical Analysis

SPSS software (16.0, IBM, USA) was used to analyze the data. When the data in multiple groups were in line with normal distribution and homogeneity of variance test, one-way analysis of variance (ANOVA) was tested, besides, the Tukey test was used for further pairwise comparison between groups. When the distribution was normal, but the variance was not uniform, Dunnett's T3 test or independent sample *t*-test was used. When it was not consistent with the normal distribution, the Kruskal-Wallis H test was used. The significance level *α* = 0.05. The data was presented as mean ± standard deviation. The level of statistical significance was defined at *P* < 0.05.

## 3. Results

### 3.1. Astaxanthin Alleviated the Pathological Change of the Intestinal Tissue in NEC Rats

**Figure 1:**
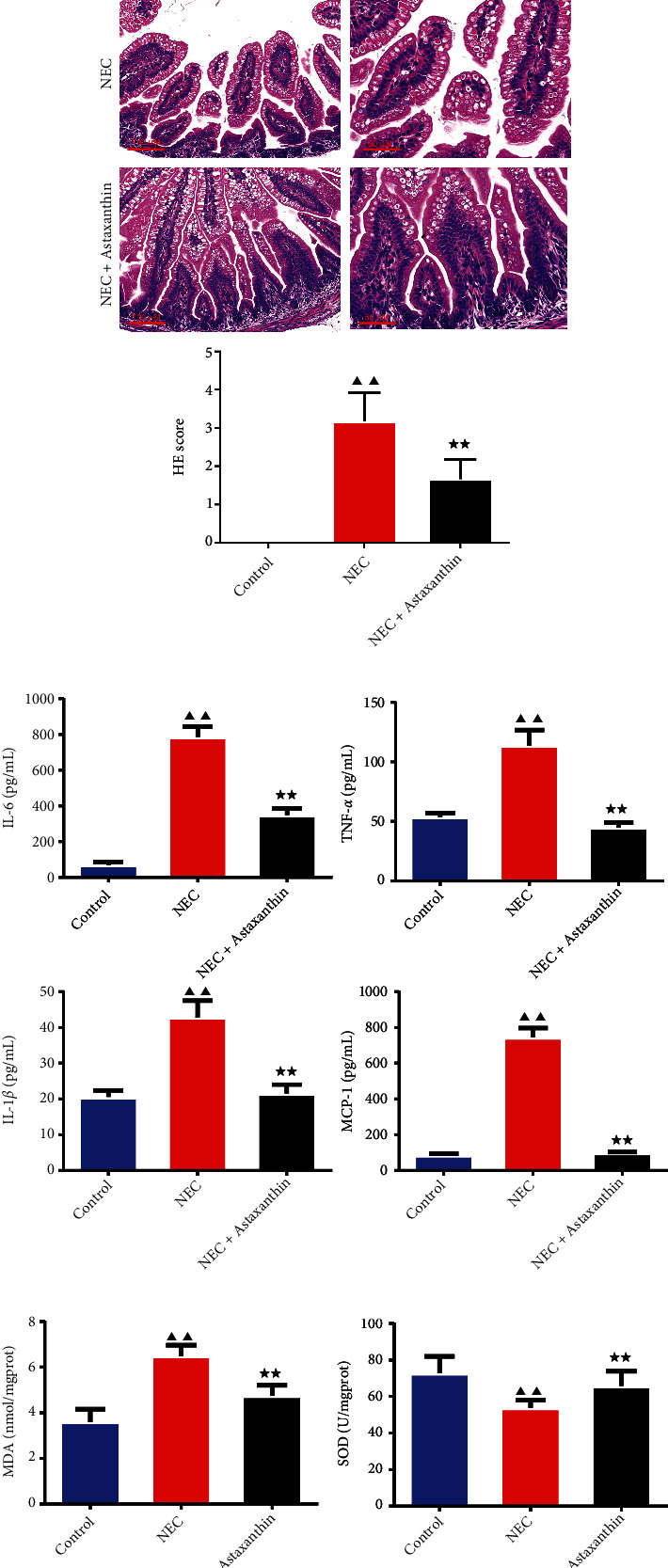
Astaxanthin alleviated the pathological change and restrained the inflammatory and oxidative stress cytokines of the intestinal tissue in necrotizing enterocolitis (NEC) rats. (a) The histomorphology in intestinal tissues in rats was observed by hematoxylin-eosin (HE) staining (magnification ×200, 400), and the HE scores were calculated in each group, *n* = 6 in each group. (b) The serum content of interleukin-1 beta (IL-1*β*), interleukin 6 (IL-6), tumor necrosis factor-*α* (TNF-*α*), and macrophage chemoattractant protein-1(MCP-1) were tested by enzyme-linked immunosorbent assay (ELISA) kit, *n* = 6 in each group. (c) The content of malondialdehyde (MDA) and superoxide dismutase (SOD) in intestinal tissue was tested by the ELISA kits. *n* = 6 in each group. ^▲▲^*P* < 0.01 vs. control group. ^★★^*P* < 0.01 vs. NEC group.

To make clear whether astaxanthin could alleviate the pathological change of the intestinal tissue in NEC rats, we conducted HE staining in each group. As is shown in Figure 1(a), the structure of intestine in control group of rats was basically normal, and the hierarchy was clear. Compared with the control group, the NEC group showed obvious inflammatory cell infiltration, lesions invaded the serosa layer, and all crypts and epithelium were destroyed. With the interference of astaxanthin, the severity of lesions in NEC rats was reduced compared with the NEC group. What's more, the semi-quantitative score of HE in the NEC + Astaxanthin group was significantly decreased compared to the NEC rats (Figure 1(a), *P* < 0.01).

### 3.2. Astaxanthin Restrained the Expressions of IL-6, IL-1*β*, TNF-*α*, and MCP-1 of Serum

To explore whether astaxanthin could suppress the inflammation response in serum, which was induced by the NEC in rats, ELISA kits were used to test the levels of IL-6, IL-1*β*, TNF-*α*, and MCP-1. As is shown in Figure 1(b), the results confirmed that after the stimulation of NEC, the levels of IL-6, IL-1*β*, TNF-*α*, and MCP-1 in serum were significantly increased compared to the control rats (*P* < 0.01). To our expectation, astaxanthin inhibited the expressions of IL-6, IL-1*β*, TNF-*α*, and MCP-1 in relative to the NEC group (*P* < 0.01).

### 3.3. Astaxanthin Suppressed MDA while Intensified SOD Expression of Intestinal Tissue in NEC Rats

With the ELISA kits, we tested the indicators of oxidative stress, including MDA and SOD. As is shown in Figure 1(c), we observed that MDA expression of intestinal tissue was higher in NEC rats than the control rats, whereas the SOD level of intestinal tissue was lower in NEC rats in contrast to control rats (*P* < 0.01). With the treatment of the astaxanthin, the content of MDA of intestinal tissue was reduced compared to NEC group, conversely, the content of SOD of intestinal tissue was enhanced (*P* < 0.01).

### 3.4. Astaxanthin Protected the Cells from Apoptosis in Intestinal Tissues of NEC Rats

**Figure 2:**
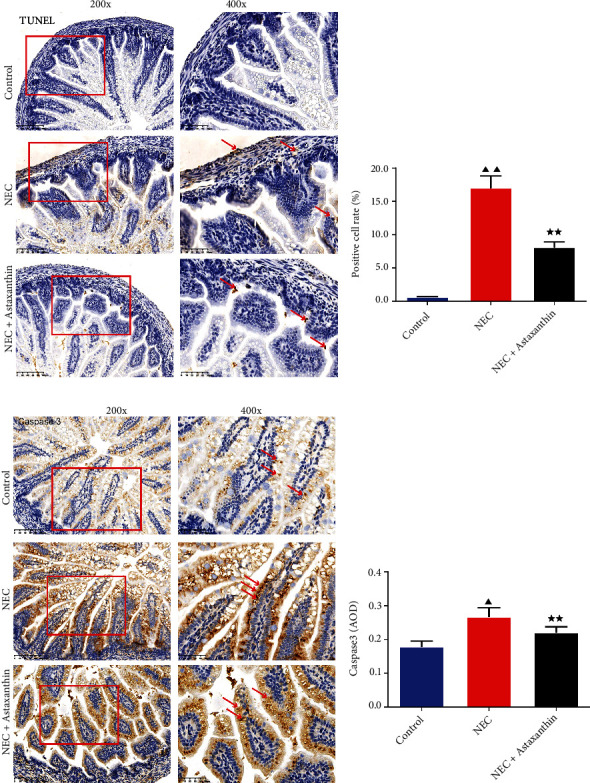
Astaxanthin protected the cells from apoptosis of intestinal tissues in NEC rats. (a and b) The apoptotic cells of intestinal tissues in rats are stained by the TUNEL kits, and the positive cell rates were calculated in each group (magnification ×200, 400), *n* = 6 in each group. (c and d) The positive expression of Caspase 3 of intestinal tissues in rats was examined by immunohistochemistry (magnification ×200, 400), and the average optical density (AOD) of the Caspase 3 was calculated in each group, *n* = 6 in each group. ^▲^*P* < 0.05, ^▲▲^*P* < 0.01 vs. control group. ^★★^*P* < 0.01 vs. NEC group.

**Figure 3:**
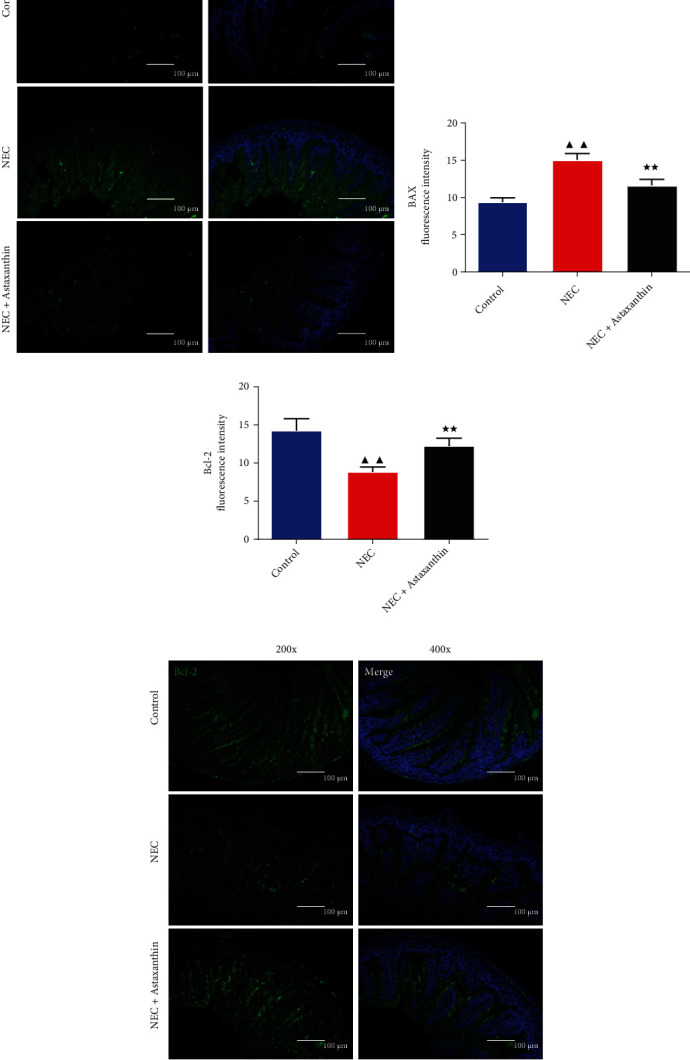
Astaxanthin reduced the expression of Bcl-2-associated X (BAX) while enhanced the expression of B-cell lymphoma-2 (Bcl-2) of intestinal tissues in NEC rats. (a, b, c, and d) Immunofluorescence staining was used to test Bcl-2, BAX expressions of intestinal tissues in rats. Magnification ×200, 400, *n* = 6 in each group; ^▲▲^*P* < 0.01 vs. control group. ^★★^*P* < 0.01 vs. NEC group.

As is shown in Figures 2(a), 2(b), 2(c), and 2(d) and Figures 3(a), 3(b), 3(c), and 3(d), with TUNEL, IHC, and immunofluorescence staining, more positive cells with apoptotic characteristics and more positive expression of Caspase 3 and higher fluorescence intensity of BAX, whereas less Bcl-2 fluorescence intensity was found of intestine tissues in NEC rats in relative to control group (*P* < 0.05 or *P* < 0.01). And with the stimulation of the astaxanthin, there were less positive cell rate with apoptotic characteristics, less Caspase 3-positive staining, fewer fluorescence intensity of BAX while the higher fluorescence intensity of Bcl-2 of intestine tissues was found than NEC group (*P* < 0.01).

### 3.5. Astaxanthin Inhibited the Inflammatory- and Apoptosis-Related Proteins of Intestinal Tissues in NEC Rats

**Figure 4:**
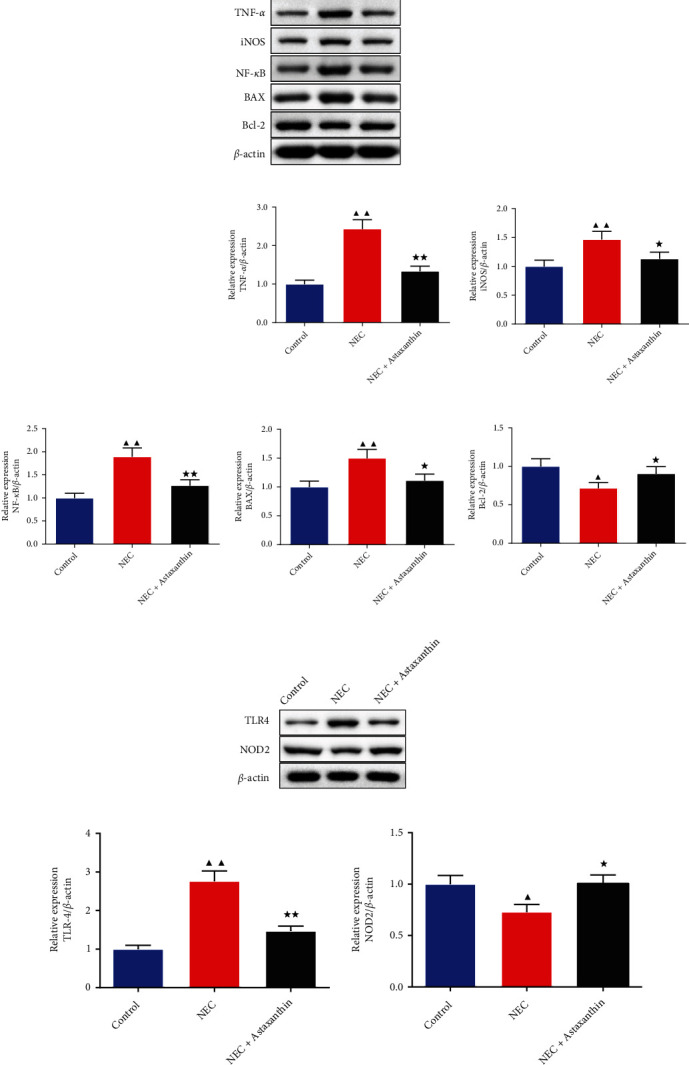
Astaxanthin inhibited the inflammatory- and apoptosis-related proteins of intestinal tissues in NEC rats. (a, b, c, and d) Western blot was used to test the expressions of TNF-*α*, inducible nitric oxide synthase (iNOS), nuclear factor-*κ*B (NF-*κ*B), BAX, Bcl-2, toll-like receptor 4 (TLR4), and nucleotide-binding oligomerization domain 2 (NOD2) of intestinal tissues in rats, *n* = 3 in each group; ^▲^*P* < 0.05, ^▲▲^*P* < 0.01 vs. control group. ^★^*P* < 0.05, ^★★^*P* < 0.01 vs. NEC group.

In Figures 4(a), 4(b), 4(c), and 4(d), we detected the expressions of inflammatory- and apoptosis-related proteins of intestinal tissues in rats with western blot assay. Results demonstrated that NEC rats had higher expression of the TNF-*α*, iNOS, NF-*κ*B, BAX, and TLR4 of intestine tissues than control rats (*P* < 0.05 or *P* < 0.01). In addition, compared with the NEC group, the protein expression levels of TNF-*α*, iNOS, NF-*κ*B, BAX, and TLR4 were significantly decreased, and the protein expression level of Bcl-2 and NOD2 was significantly increased in NEC + Astaxanthin group (*P* < 0.05 or *P* < 0.01).

### 3.6. NOD2 Inhibitor Offset the Protective Effect of Astaxanthin on Pathological Change of Intestinal Tissues in NEC Rats

**Figure 5:**
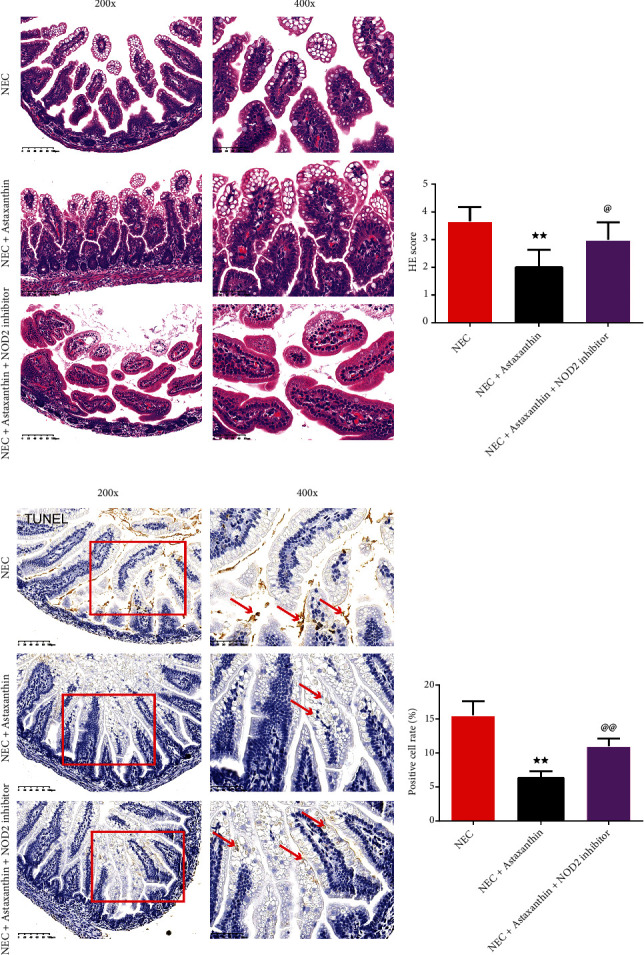
NOD2 inhibitor offset the protective effect of Astaxanthin on intestinal tissues in NEC rats. (a and b) The histomorphology in intestinal tissues in rats was observed by HE staining (magnification ×200, 400), and the HE scores were calculated in each group, *n* = 6 in each group. (c and d) The apoptotic cells of intestinal tissues in rats are stained by the TUNEL kits, and the positive cell rates were calculated in each group (magnification ×200, 400), *n* = 6 in each group, ^★★^*P* < 0.01 vs. NEC group, ^@^*P* < 0.05, ^@@^*P* < 0.01 vs. NEC + Astaxanthin group.

As shown in Figure 5(a), in NEC group and NEC + Astaxanthin + NOD2 inhibitor group, inflammatory cell infiltration, crypt, villi, and other tissue structures were destroyed, and the damage was severe. Compared with NEC group, NEC + Astaxanthin group showed a reduction in the degree of intestinal lesion injury. In Figure 5(b), compared with NEC group, HE score of intestinal tissue of rats in NEC + Astaxanthin group was significantly decreased (*P* < 0.01). And the HE score of intestinal tissue in NEC + Astaxanthin + NOD2 inhibitor group was significantly higher than NEC + Astaxanthin group (*P* < 0.05).

### 3.7. NOD2 Inhibitor Offset the Protective Effect of Astaxanthin Reducing Apoptosis Cells of Intestinal Tissues in NEC Rats

As is shown in Figures 5(c) and 5(d), with TUNEL staining, astaxanthin reduced the percentage of the positive cell rate of intestinal tissues in relative to the NEC rats (*P* < 0.01). However, this trend was reversed of intestinal tissues by the addition of the NOD2 inhibitor on the NEC + Astaxanthin rats (*P* < 0.01).

### 3.8. Astaxanthin Exerts Protective Effect by Activating NOD2 and Inhibiting TLR4 Signaling Pathway on Intestinal Tissues in NEC Rats

**Figure 6:**
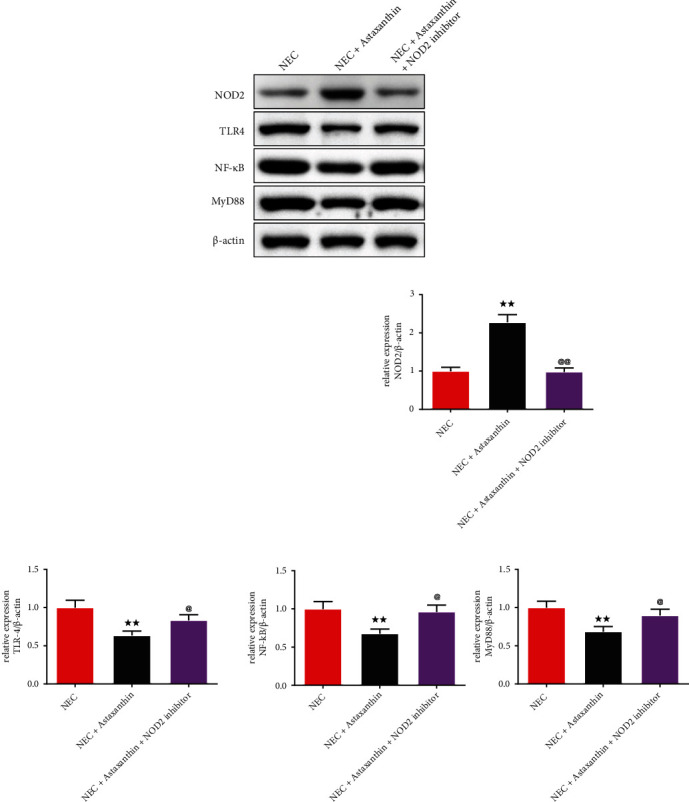
Astaxanthin exerts protective effect by activating NOD2 and inhibiting TLR4 signaling pathway on intestinal tissues in NEC rats. (a and b) Western blot was used to test the expressions of NOD2, TLR4, NF-kB, and MyD88 of intestinal tissues in rats, *n* = 3 in each group; ^★★^*P* < 0.01 vs. NEC group, ^@^*P* < 0.05, ^@@^*P* < 0.01 vs. NEC + Astaxanthin group.

In Figures 6(a) and 6(b), western blot was used to test the expression of the NOD2 and the TLR4 pathway-related proteins. The results demonstrated that in the NEC + Astaxanthin group, the expression of the NOD2 was strengthened, whereas the expressions of the TLR4, NF-*κ*B, and MyD88 were suppressed on intestinal tissue in relative to the NEC rats (*P* < 0.01). After adding the NOD2 inhibitor on the NEC + Astaxanthin group, the NOD2 expression in intestinal tissues was reduced but the expressions of the TLR4, NF-*κ*B, and MyD88 in intestinal tissues were elevated in relative to the NEC + Astaxanthin group (*P* < 0.05 or *P* < 0.01).

## 4. Discussion

NEC is a neonatal gastrointestinal tract illness, with inflammation and bacterial invasion, that causes severe neonatal morbidity and mortality [18]. It has been reported that astaxanthin is a carotenoid with strong antioxidant and anti-inflammatory activities [19]. The present study suggested that astaxanthin reduces oxidative stress, inflammatory response, and cell apoptosis in NEC rats by enhancing NOD2 to inhibit TLR4 pathway.

Through the peroxidation of unsaturated lipids, which causes intestinal hypoxia/reoxygenation damage, oxidative stress has been linked to the pathophysiology of NEC [20]. However, thanks to the lipophilic characteristics of astaxanthin, it enables to penetrate the cell double layer membrane and realize its antioxidant effect. The studies discovered that astaxanthin attenuates serious diseases from oxidative stress-induced dysfunction [21–23]. The present study proved that astaxanthin reduced oxidative stress by downregulating MDA but upregulating SOD levels in intestinal tissue, which thus contributed to the alleviation effect on intestinal injury in NEC neonatal rats.

Moreover, inflammation is crucial in the progression of NEC. Cytokines cause the NEC's mucosal damage to rise during inflammation [24]. TNF-expression has been linked to increased chemokine and cytokine production in the intestines of NEC patients [25, 26]. Consistent with the previous study, we found that in NEC rats, the inflammation cytokines emerged, such as the elevation of the TNF-*α*, iNOS, etc. Nevertheless, we discovered that astaxanthin successfully inhibits the inflammation response in NEC rats. Apart from that, NEC is characterized by extensive necrosis and apoptosis of the intestinal epithelium [27, 28]. In our study, with the TUNEL staining, we found astaxanthin manages to protect cells from apoptosis. In addition, the expressions of the BAX and Caspase3 were produced less after the astaxanthin treatment, whereas more Bcl-2 was produced in intestinal tissue of NEC rats, indicating that astaxanthin exerted the protective effect in NEC rats. Akduman et al. [29] declared that astaxanthin has the capability to minimize the extent of intestinal impairment in NEC due to its anti-inflammatory, antioxidant, and other beneficial effects, which was consistent with our research.

Most importantly, in our study, astaxanthin upregulated the expression of the NOD2, which was lower expressed in the NEC rats, meanwhile, it downregulated the expression of the TLR4, NF-kB, and MyD88. According to the previous study of Richardson et al. [8], they revealed that NOD2 inhibits TLR4 signaling in the intestinal epithelium *in vivo* and *in vitro* experiments with lower NOD2 and more TLR4 receptors, which could explain the change of the NOD2 and TLR4 in NEC rats in our study. And we found that the protective effect was offset by NOD2 inhibitor addition with a worse pathological change of the intestinal tissue and more apoptosis cells, further substantiating that the astaxanthin enhanced the expression of the NOD2 to inhibit TLR4 signal pathway to exert function in the NEC rats. The likely explanation for astaxanthin towards the interaction of the NOD2 and TLR4 receptors is that astaxanthin activating NOD2 may contribute to the activation of inflammatory vesicles in enterocytes, thereby alerting the host to the presence of invasion by establishing a pro-inflammatory response while limiting the extent of TLR4 pathway. In addition, it effectively prevents further bacterial entry, while also limiting the impact of TLR4-induced intestinal injury [30].

In this study, the treatment of astaxanthin reduces intestinal tissue damage in NEC neonatal rats by alleviating oxidative stress, inflammatory response, and cell apoptosis. However, the inner crosstalk between NOD2 and TLR4 needed to be further probed and cell experiments verification of astaxanthin-specific efficacy in the prevention and treatment of NEC is needed.

## 5. Conclusion

In conclusion, astaxanthin improved the pathological damage of the intestinal tissue in NEC rats, by reducing the oxidative stress with lower MDA but higher SOD level and protects cells from apoptosis of intestinal tissues in NEC neonatal rats. Moreover, it inhibited the inflammatory response that less TNF-*α*, iNOS, TLR4, NF-*κ*B, and MyD88 were produced, but enhanced NOD2 expression. NOD2 inhibitor offset the protective effect of the astaxanthin on the NEC rats. Astaxanthin alleviated oxidative stress, inflammatory response, and apoptosis in neonatal NEC rats by enhancing NOD2 and inhibiting the TLR4 pathway.

## Data Availability

Data from this study can be obtained from co-author upon request.

## Abbreviation

NEC: Necrotizing enterocolitis
SOD: Superoxide dismutase
MDA: Malondialdehyde
TNF-*α*: Tumor necrosis factor-*α*
IL-1*β*: Interleukin-1*β*
IL-6: Interleukin 6
MCP-1: Macrophage chemoattractant protein-1
iNOS: Inducible nitric oxide synthase
NF-*κ*B: Nuclear factor-*κ*B
Bcl-2: B-cell lymphoma-2
BAX: Bcl-2-associated X
TLR4: Toll-like receptor 4
NOD2: Nucleotide-binding oligomerization domain 2
ELISA: Enzyme-linked immunosorbent assay
H&E: Hematoxylin-eosin
IHC: Immunohistochemistry.
